# Follow-up Findings in a Turkish Girl with Pseudohypoparathyroidism Type Ia Caused by a Novel Heterozygous Mutation in the GNAS Gene

**DOI:** 10.4274/jcrpe.3191

**Published:** 2017-03-01

**Authors:** Sezgin Şahin, Olaf Hiort, Susanne Thiele, Olcay Evliyaoğlu, Beyhan Tüysüz

**Affiliations:** 1 İstanbul University Cerrahpaşa Faculty of Medicine, Department of Pediatric Rheumatology, İstanbul, Turkey; 2 University of Lübeck, Department of Pediatrics, Lübeck, Germany; 3 İstanbul University Cerrahpaşa Faculty of Medicine, Department of Pediatric Endocrinology, İstanbul, Turkey; 4 İstanbul University Cerrahpaşa Faculty of Medicine, Department of Pediatric Genetics, İstanbul, Turkey

**Keywords:** Pseudohypoparathyroidism Ia, Albright hereditary osteodystrophy, ectopic ossification, GNAS gene, Gsα activity, short stature

## Abstract

Pseudohypoparathyroidism type Ia (PHP-Ia) is characterized by multihormone resistance and an Albright hereditary osteodystrophy (AHO) phenotype. It is caused by heterozygous mutations in *GNAS* gene. Clinical and biochemical findings of a female PHP-Ia patient were evaluated from age of diagnosis (6.5 years) to 14.5 years of age. The patient had short stature, brachydactyly, and subcutaneous heterotopic ossifications. Serum calcium and phosphorus levels were normal, but parathyroid hormone levels were high. Based on the typical clinical findings of AHO phenotype and biochemical findings, she was diagnosed as having PHP-Ia. A novel heterozygous mutation (c.128T>C) was found in the *GNAS* gene. Follow-up examinations revealed resistance to thyroid-stimulating hormone and a bioinactive growth hormone. Clinicians should take into consideration PHP-Ia in patients referred with short stature, and patients with an AHO phenotype must be further evaluated for hormone resistance, *GNAS* gene mutation, Gsα activity. To our knowledge, this is the first case report describing bioinactive growth hormone in PHP-Ia.

**WHAT IS ALREADY KNOWN ON THIS TOPIC?**

Pseudohypoparathyroidism type Ia (PHP-Ia) is characterized by multihormone resistance and Albright hereditary osteodystrophy. Heterozygous mutations in GNAS gene may result with decreased Gsα activity.

**WHAT THIS STUDY ADDS?**

Longitudinal follow-up of a PHP-Ia patient seems to be lacking in the literature. In our case, heterotopic ossification, subclinical hypothyroidism, and cerebral calcification developed late during the 8-year follow-up period. To our knowledge, this is the first report about bioinactive growth hormone associated with PHP-Ia. GNAS gene analysis revealed a novel mutation.

## INTRODUCTION

Pseudohypoparathyroidism (PHP) is defined as an end-organ resistance to parathormone (PTH) and is characterized by hypocalcemia, hyperphosphatemia, and increased PTH levels (1).

PHP-Ia is a subtype of PHP, caused by heterozygous inactivating mutations in *GNAS* which encodes Gsα. This gene is located on chromosome 20q13.11 and contains 13 exons and 12 introns. Gsα is essential for the actions of PTH and of many other hormones (2,3). PHP-Ia patients express resistance to hormones that act via Gs-coupled receptors, such as PTH, thyroid-stimulating hormone (TSH), gonadotropins, growth hormone-releasing hormone (GHRH). These patients, also known as cases of Albright hereditary osteodystrophy (AHO), show a constellation of features including short stature, short neck, round face, centripedal obesity, brachydactyly, ectopic ossifications, and mild mental retardation.

Low serum calcium, elevated serum phosphorus and high PTH are frequent findings in laboratory analyses. In some cases, calcium and phosphorus are within the normal range and only PTH is elevated (2,4).

The differential diagnosis among subtypes of PHP is very difficult. Table 1 shows subtypes of PHP with the classification based on the level of serum calcium, phosphorus, PTH, urinary cyclic AMP (cAMP), phosphaturia response to PTH, Gsα activity, and presence of AHO phenotype. PHP-I can be differentiated by the presence (PHP-Ia and PHP-Ic) or absence (PHP-Ib) of AHO. The only way to distinguish PHP-Ia from the much rarer PHP-Ic is to measure Gsα activity because of the identical clinical and biochemical features.

In this case report, we present the follow-up results of a longitudinal observation of clinical and biochemical profiles of a girl with typical phenotype of AHO and a novel mutation in *GNAS* gene.

## CASE REPORT

**Figure 1:**
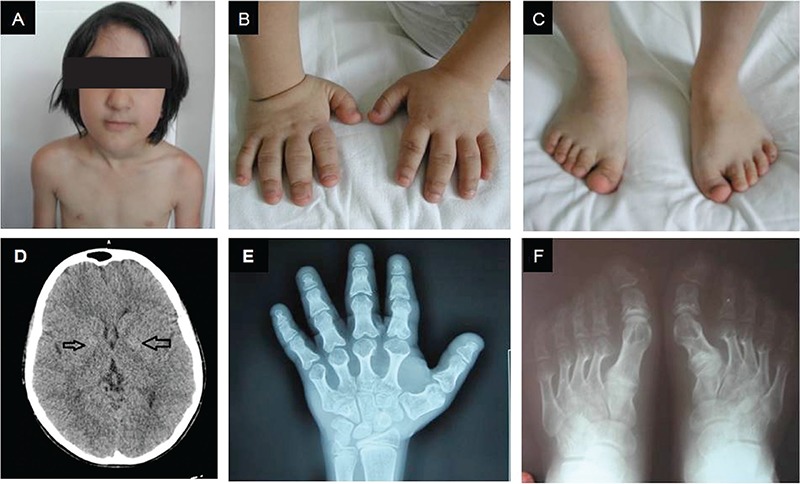
The photograph of the patient at age 7 years. Note round face, full cheeks, and short neck (A). Short hands and feet (B, C). Cranial computerized tomography showing bilateral calcification of globus pallidus (D). Roentgenogram of hands and feet. Marked shortness of metacarpals and metatarsals (especially 4^th^ and 5^th^). (E, F) Cone-shaped epiphyses are visible in all tubular bones of hand

This 6.5-year-old girl patient was referred to our outpatient clinic for short stature and brachydactyly. She was born at term to non-consanguineous parents. Neither brachydactyly, short stature, nor any specific feature of AHO phenotype were present in either parent. The patient’s birth weight was 2300 g (<10 p) and her birth height was 45 cm (<10 p). Developmental dysplasia of the hip was noted on neonatal examination. Her motor development was also delayed. She was reported to first smile to her mother at 3 months, to have acquired head control at 9 months, sitting at 24 months, walking at age 3 years. Physical examination revealed short stature [height: 109 cm, -1.75 standard deviation (SD)], a low body mass index (BMI=13.5 kg/m^2^, -1.59 SD), round face, full cheeks, depressed nasal bridge, short neck, brachydactyly of all digits of the hand (hand length: 9.5 cm, <3^th^ centile) and feet (Figure 1 A-1C and Table 2). Subcutaneous heterotopic ossification at the level of the right iliac crest (diameter 3x2 cm) was also noted.

The first laboratory examination revealed normal serum calcium (9.7 mg/dL) and phosphorus (5.3 mg/dL) levels, with an elevated serum PTH (138.1 pg/mL) level. The subsequent measurements of PTH concentrations were 98.5 pg/mL and 111.2 mg/mL, respectively. The serum levels of free triiodothyronine (fT_3_), free thyroxine (fT_4_), TSH, alkaline phosphatase (ALP), creatinine, and 25-hydroxyvitamin D (25-OHD) were normal. X-rays revealed marked shortness and thickness of metacarpal and metatarsal bones with cone-shaped epiphyses in all tubular bones of the hand (Figure 1E, 1F) and coxa valga with acetabular dysplasia. The patient’s bone age was compatible with her chronological age. Cranial magnetic resonance imaging findings were normal. Chromosomal analysis revealed 46,XX karyotype. Audiological and ophthalmological examination showed bilateral minimal conductive hearing loss and retinitis pigmentosa, respectively. Gsα function was found as 48.1% of normal, suggesting impaired activity. Based on these clinical and biochemical findings, the most likely diagnosis was thought to be PHP-Ia. A heterozygous novel missense mutation (c.128T>C) was detected in exon 1 in the GNAS gene.

The patient was followed up until 14.5 years of age. At age 9 years, her height was 122 cm (-1.86 SD) and her BMI was 14.8 kg/m^2^ (-0.86 SD) (Table 2). Borderline intellectual disability (IQ: 83) was detected in Stanford-Binet test at age 9 years. Pubertal development was Tanner stage II and appropriate to her age. Hormonal profile showed subclinical hypothyroidism (fT_3_: 3.51 pg/mL, fT_4_: 1.27 ng/dL, TSH: 5.97 mIU/L). [The diagnosis of subclinical hypothyroidism was based on a serum TSH value of >4.2 µU/mL (reference interval=0.27-4.2 µU/mL), while serum fT_3_ (reference interval=2-4.4 pg/mL) and fT_4_ (reference interval=0.93-1.7 ng/dL) levels were within the reference ranges].

Antithyroid antibodies were within normal limits and thyroid ultrasound was normal. Thus, increased TSH (6.02 mIU/L) with normal fT_3_ and fT_4_ levels in subsequent measurements suggested TSH resistance. Elevated PTH levels (138.1 pg/mL) were still evident, while serum calcium, phosphorous, and ALP concentrations were normal. Nephrocalcinosis was not detected in ultrasonography. Cranial computerized tomography (CT) scan did not reveal any basal ganglion calcification. L-T4 supplementation was initiated at a dose of 1.25 µg/kg/day.

The most remarkable findings of the physical examination at age 14.5 years were the increase in number of mobile subcutaneous heterotopic ossifications and bilateral calcification of the globus pallidus in cranial CT scan (Figure 1D). In addition to the initial lesion at the right iliac crest level, there were three new subcutaneous ossifications in the both hands and in the left foot. Her height was 143 cm (-2.80 SD) and her weight was 35 kg (-2.54 SD) (Table 2). While pubertal development was at Tanner stage IV, menarche had not occurred yet. During the 8 years of longitudinal follow-up, her pubertal development had been normal, indicating that there was no gonadotropin resistance. The patient’s bone age was 13 years. Her height standard deviation score (SDS) regressed to -2.80 with a growth velocity of 5 cm/year. Growth hormone (GH) stimulation tests were performed. They revealed sufficient GH secretion (after clonidine stimulation GH peak: 14 ng/mL). Insulin-like growth factor 1 (IGF-1) stimulation test was performed to see if there was a response of IGF-1 increment to exogenous GH. This test revealed an increased response [baseline IGF-1 level: 262.1 ng/mL (-2.086 SD), peak IGF1 level: 393.5 ng/mL, ΔIGF1: 131.4 ng/mL, 50.1% increase] suggesting GH bioinactivity. [The IGF-1 generation test was performed as follows: Exogenous GH injections (100 µg/kg s.c. daily) were administered at 21:00 h for four days. Blood samples for IGF-1 were taken on day 0 before the first injection and 12 hours later after the last injection (a serum IGF-1 level increment greater than 20% was defined as GH bioinactivity). Baseline serum IGF-1 standard deviation calculation was performed (5)].

### Molecular Methodology

The activity of Gsα protein from erythrocyte membranes of patients was investigated in heparinized blood samples. After solubilization, the Gsα protein from patient-derived erythrocyte membranes was incubated with GTPγS. Adenylyl cyclase from turkey red cell membranes were added, and the generated cAMP in the presence of ATP by RIA (Immuno Biological Laboratories, Hamburg, Germany) was measured. Results obtained in triplicate were expressed as percent of the mean of healthy controls (normal range: 85-115%).

For molecular genetic analysis, genomic DNA derived from peripheral leukocytes was isolated by standard procedures (Qiaquick DNA kit, Qiagen, Hilden, Germany). GNAS exon 1-13, (RefSeq NM_000516.4) including all intron/exon boundaries were amplified in 11 fragments by polymerase chain reaction (PCR) (primer sequences available upon request). PCR-amplified DNA was sequenced by direct cycle sequencing using the BigDye Terminator v1.1 Cycle Sequencing Kit (Applied Biosystems, Foster City, CA) and an ABI 3130 capillary sequencer (Applied Biosystems, Foster City, CA).

## DISCUSSION

The hand and feet X-rays of this patient (clinical findings: short stature, short neck, round face, brachydactyly, and borderline intellectual disability) revealed marked shortness of metacarpals and metatarsals. The patient was also found to have high PTH levels, subclinical hypothyroidism, and heterotopic ossifications. While patients with AHO are usually obese, the BMI level of our patient was in the underweight group according to the World Health Organization guidelines. With these findings, the patient was diagnosed as a case of Albright PHP. Heterotopic ossifications and brachydactyly are the most unique features of AHO phenotype that distinguishes true AHO from a variety of clinical phenocopies (6,7). While brachydactyly was evident in the first evaluation of our patient, heterotopic ossification became apparent at the right iliac crest at 9 years of age. Moreover, the subcutaneous ossifications were observed to have increased in number at different parts of the body in the last follow-up examination.

Almost all features of PHP-Ia including hormone resistance, are also common in acrodysostosis syndrome. PHP-Ia can be differentiated from this syndrome only by the presence of GNAS mutation and of heterotopic ossifications (8). The differential diagnosis among subtypes of PHP is very difficult. In our patient, PHP-Ib and PHP-Ic were excluded by decreased Gsα activity and PHP-II by both decreased Gsα activity and mutation. Gsα activity of our case was reduced to 48.1%, a finding that was consistent with PHP-Ia rather than PHP-Ib, -Ic, and -II.

The *GNAS* gene analysis revealed a novel heterozygous mutation (c.128T>C). This mutation results in the change of the aminoacid leucine at codon 43 with the aminoacid proline (p. Leu43Pro). There is a moderate physicochemical difference between Leu and Pro [Grantham dist.: 98 (0-215)] and the amino acid leucine at codon 43 is conserved between species up to C. elegans. It is possible that the mutation is pathogenic and causal for AHO in this patient. This mutation, as far as we know, has never been described before and was not detected in the *GNAS* gene. Thus, we have concluded that the mutation is meaningful.

Formerly, the term pseudopseudohypoparathyroidism (PPHP), was used for patients who display AHO features and carry heterozygous inactivating Gsα mutations without evidence of hormone resistance. Previous studies were reporting maternal inheritance of GNAS mutations results in AHO together with hormone resistance and named as PHP-Ia, while paternal inheritance of the same mutation was reported to lead only to AHO phenotype and was termed as PPHP (2,3). However, in a recent publication, mild PTH resistance besides AHO phenotype was reported in a PPHP patient, and ascertainment of the parental origin of the mutation was declared as the most effective diagnostic procedure in differentiating PPHP from PHP-Ia (9).

While Gsα is biallelically expressed in most tissues, it is predominantly maternally expressed in certain tissues such as renal proximal tubules, the thyroid, the gonads, and the pituitary. Paternal allele is suppressed in these tissues. Tissue-specific imprinting nature of GNAS gene is responsible for this difference (1,10). This might explain why the multi-hormone resistance in PHP-Ia patients primarily involves four hormones: PTH, TSH, gonadotropins, and GHRH (1,11).

While PTH resistance associated later with TSH resistance was apparent in our patient, resistance to gonadotropins or GHRH was not detected. The most important hormone resistance in PHP-Ia that results in clinically evident signs is renal resistance to PTH. Most patients present with hypocalcemia, hyperphosphatemia, and elevated levels of PTH. Despite presence of PTH resistance, some cases may have normal serum calcium and phosphorus levels. PHP-Ia patients have a reduced phosphatidic response to PTH, which leads to hyperphosphatemia. Besides hyperphosphatemia, proximal tubule resistance to PTH leads to decreased 1,25-dihydroxyvitamin D production, thus causing hypocalcemia. Unlike patients with primary hypoparathyroidism, PHP cases do not develop hypercalciuria, which shows that the anti-calciuric effect of PTH in the thick ascending limb remains intact (3). Some patients may show osteopenia and signs of rickets. Skeletal deformities like short ulna, genu varum-valgum, cubitus valgus may be seen (12).

TSH resistance becomes clinically apparent during the adolescence period (1). This resistance is mostly not severe, with TSH levels only slightly elevated or thyroid hormone levels slightly less than normal (11). Our patient developed subclinical hypothyroidism at 9 years of age.

Although short stature became apparent in the follow-up of our patient, she showed a normal GH response to clonidine stimulation test. Clonidine is a selective α-receptor agonist and causes GH release via GHRH. This normal response suggests that our patient did not have GHRH resistance. GH bioinactivity was diagnosed by detecting a 50.1% increment to IGF1 stimulation test. This diagnosis predicates that endogenously produced GH is inactive, probably due to disorders of GH gene. To our knowledge, this is the first report about the bioinactive GH associated with PHP-Ia. However, without GH gene analysis, this diagnosis is not precise. Reports on GH deficiency in patients with PHP-Ia are variable. While some authors reported GH deficiency in PHP-Ia (11,13,14,15), there are a few studies reporting patients without GH deficiency (16,17) as noted in the case of our patient.

However, our inability to perform a PTH infusion test and a *GNAS* gene analysis in the parents represent the main limitations of this case report, limitations which made the differential diagnosis more complicated in terms of PHP-Ia and PPHP. Also, we had no possibility to analyze neither the DNA samples of the parents nor the ribonucleic acid sample of the patient to be able to show the origin of the mutation, in other words, whether it was paternally or maternally expressed. Accordingly, the patient could also be classified as PPHP in the light of current literature and case reports.

In conclusion, clinicians should take into consideration PHP-Ia and PPHP in patients referred with short stature, and the subjects with AHO phenotype must be further evaluated for hormone resistance, *GNAS* gene mutation, Gsα activity. Repeated physical and laboratory examinations should be performed in order to detect changes which may occur in hormone resistance.

## Figures and Tables

**Table 1:**
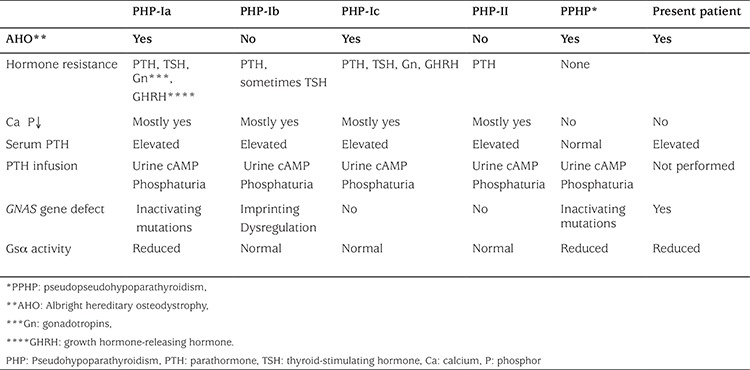
Clinical findings of our patient and differential diagnosis in cases of pseudohypoparathyroidism

**Table 2:**
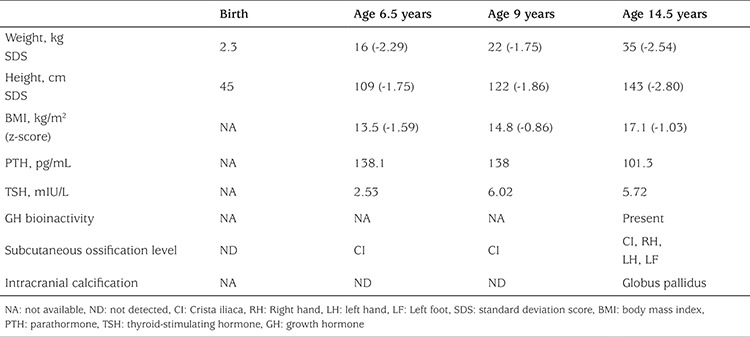
Results of anthropometry and hormone measurements during the follow-up of the patient
